# NIR-Mediated drug release and tumor theranostics using melanin-loaded liposomes

**DOI:** 10.1186/s40824-022-00270-w

**Published:** 2022-06-03

**Authors:** Min Ah. Kim, Chang-Moon Lee

**Affiliations:** 1 Department of Biomedical Engineering, Chonnam National University Graduated School, Yeosu, Jeonnam 59626 Republic of Korea; 2 School of Healthcare and Biomedical Engineering, Chonnam National University, Yeosu, Jeonnam 59626 Republic of Korea; 3 Research Center of Healthcare Biomedical Engineering, Chonnam National University, Yeosu, Jeonnam 59626 Republic of Korea

**Keywords:** Melanin, Perfluorohexane, Liposome, Photothermal cancer therapy, Ultrasound imaging

## Abstract

**Background:**

Heat generation in a drug delivery carrier by exposure to near-infrared (NIR) light with excellent tissue transmittance is an effective strategy for drug release and tumor therapy. Because liposomes have amphiphilic properties, they are useful as drug carriers. Liposomes are also very suitable for drug delivery strategies using heat generation by NIR laser because lipid bilayers are easily broken by heat. Thermally generated bubbles from liposomes not only induce drug release, but also enable ultrasound imaging.

**Methods:**

Melanin, perfluorohexane (PFH), and 5-fluorouracil (5-FU)-loaded liposomes (melanin@PFH@5-FU-liposomes) that can generate heat and bubble by NIR laser irradiation were prepared by a thin film method. Conversion of light to heat and bubble generation of melanin@PFH@5-FU-liposomes were evaluated using an infrared (IR) thermal imaging camera and an ultrasound imaging system both *in vitro* and *in vivo*. To investigate tumor therapeutic effect, NIR laser of 808 nm was used to irradiate tumor site for 10 min after injecting melanin@PFH@5-FU-liposome into tail veins of CT26-bearing mice.

**Results:**

Melanin@PFH@5-FU-liposomes showed a spherical shape with a size of 209.6 ± 4.3 nm. Upon NIR laser irradiation, melanin@PFH@5-FU-liposomes exhibited effective temperature increase both *in vitro* and *in vivo*. In this regard, temperature increase caused a phase transition of PFH to induce bubble generation dramatically, resulting in effective drug release behavior and ultrasound imaging. The temperature of the tumor site was increased to 52 t and contrast was greatly enhanced during ultrasound imaging due to the generation of bubble. More importantly, tumor growth was effectively inhibited by injection of melanin@PFH@5-FU-liposomes with laser irradiation.

**Conclusions:**

Based on intrinsic photothermal properties of melanin and phase transition properties of PFH, melanin@PFH@5-FU-liposomes exhibited effective heat and bubble generation upon NIR laser irradiation. The elevated temperature induced bubble generation, resulting in contrast enhancement of ultrasound imaging. Melanin@PFH@5-FU-liposomes under NIR laser irradiation induced the death of cancer cells, thereby effectively inhibiting tumor growth. These results suggest that melanin@PFH@5-FU-liposomes can be utilized as a promising agent for photothermal tumor therapy and ultrasound imaging.

## Introduction

Although incision surgery is being actively performed as a representative treatment for cancer, there is a possibility of damaging nearby healthy organs. In addition, it is difficult to apply incision surgery to tumors discovered late. Moreover, monotherapy including surgery cannot achieve satisfactory therapeutic effect in clinical practice due to tumor heterogeneity and complexity [1]. In this regard, although chemotherapy and radiation therapy have been used in combination to treat cancer, their therapeutic efficiency is very low with many side effects. Therefore, it is necessary to develop a new strategy that can maximize the therapeutic effect and minimize side effects by delivering anticancer drugs to tumor site or by integrating laser or radiation into the tumor site.

Photothermal therapy (PTT) has recently attracted attention for treating cancer or inflammation. When PTT is applied to treat cancer tissues, a photosensitizer accumulated in the cancer tissue can generate heat after exposure to a laser. Cancer cells that are relatively vulnerable to heat will die [2]. Cancer cells have a lower resistance to heat than normal cells because they have an abnormal heat release mechanism due to abnormal angiogenesis [3]. For this reason, it is possible to kill cancer cells non-invasively and selectively by generating a local high-temperature condition around cancer tissues. In addition, the generated heat can control the release of drugs from nanocarriers, maximizing the efficacy of chemotherapy. The most important points to consider for successful PTT are the development of a photosensitizer (PS) with excellent biocompatibility and permeability of laser into target tissues [4].

Most conventional PSs for PTT have been developed through chemical synthesis. There are concerns about the safety of PSs due to the possibility of inducing toxicity. In addition, PSs could precipitate and aggregate easily in solution, causing blood clots in the body and reducing the activity of photothermal agents due to photobleaching [5, 6]. For this reason, there are very few cases in which PSs for PTT are applied clinically. Therefore, it is necessary to develop a natural PS that does not cause toxicity *in vivo* with excellent photo-characteristics.

Melanin is a pigment that determines the color of the skin produced and decomposed in the body of animals [7]. Melanin has excellent biocompatibility and biodegradability, which satisfies the essential conditions for PSs. Its utility value is very high [8]. In particular, melanin can absorb light of all wavelengths, especially near-infrared rays, thus generating heat effectively [9]. Many attempts have been made to utilize melanin in biomedical fields including PTT [10–12]. Melanin-based nanoparticles (MNPs) are wonderfully applied to the field of phototherapy, biological imaging, antioxidant therapy, as well as drug delivery system [13, 14]. Not only that, MNPs can modulate an immune response [15]. Li et al. reported that a photothermal and immune co-therapy strategy based on MNPs exhibited superior performance for treating primary and abscopal breast cancers [16]. Furthermore, research combining PTT using melanin and a triggered drug delivery system that can induce drug release in an instant by elevated heat is being actively conducted [17].

Recently, bubble-generating nanoparticles above specific temperature have been proposed to control drug release and provide ultrasound imaging [18–20]. Among many bubble-generating materials, perfluorohexane (PFH) belonging to perfluorocarbon (PFC) family has a vapor pressure of 27 kPa at 25°C and a boiling point of 56°C. PFH can be converted to a gas when a phase transition occurs, leading to drug release from nanocarriers. It can be used as an ultrasound imaging agent [21–23]. In detail, when PFH is encapsulated in a drug carrier containing melanin, upon application of light in the near-infrared region, bubble formation from PFH is promoted by light to perform heat conversion by melanin, resulting in explosive drug release and ultrasound imaging.

**Fig. 1:**
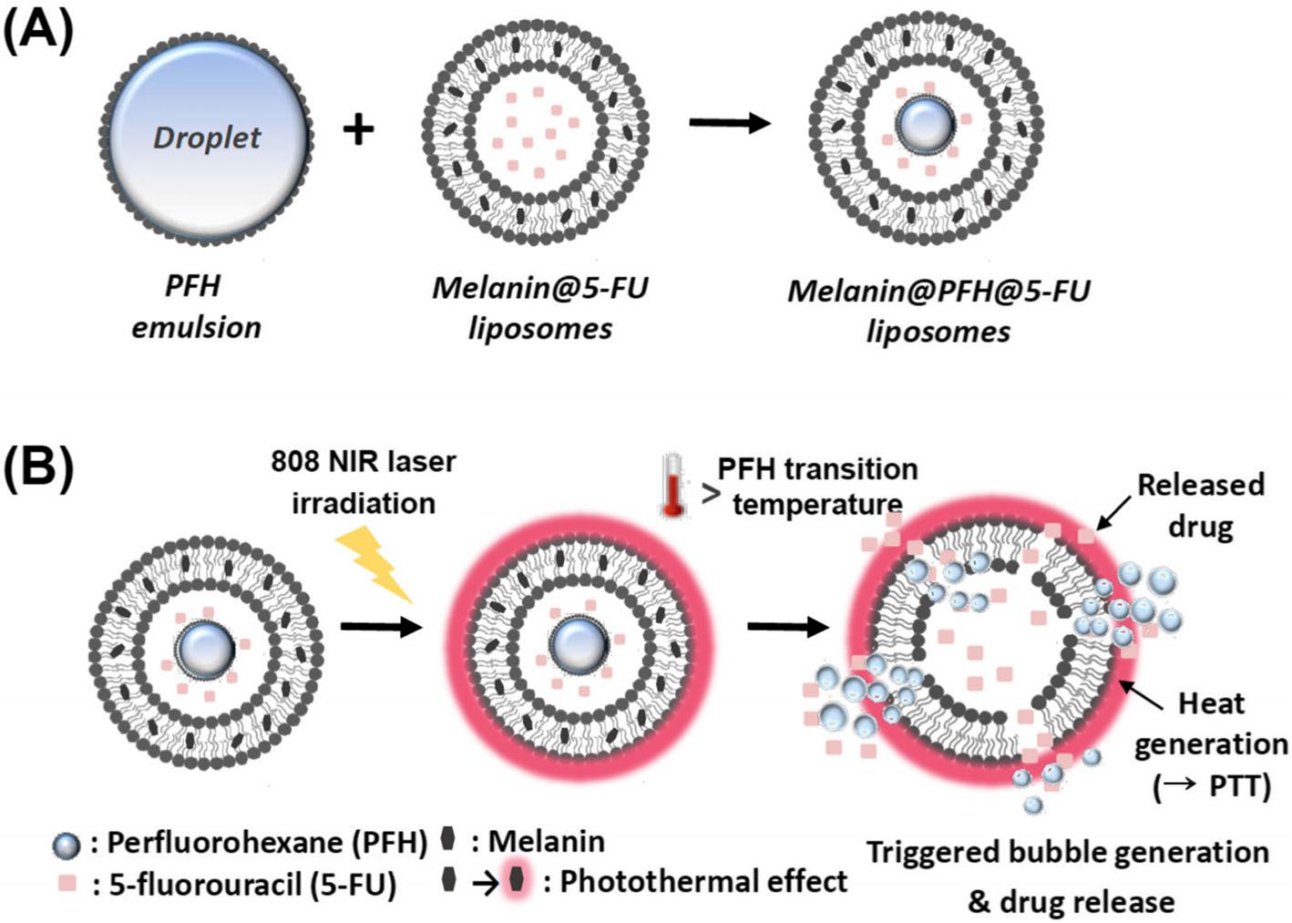
Schematic illustration of (**A**) the preparation of melanin@PFH@5-FU-liposome and (**B**) when melanin@PFH@5-FU-liposome was irradiated with an 808 nm NIR laser at a power density of 1.5 W/cm^2^, the photothermal effect of melanin induced vaporization of PFH and accelerates 5-FU release

In the present study, liposomes containing melanin and PFH are proposed for controlled release of 5-fluorouracil (5-FU) and ultrasound imaging of the lesion through a sequential process of NIR laser-responsive photothermal bubble generation (Fig. 1). Liposomes, known as representative biocompatible drug carriers, can easily encapsulate drugs and undergo surface modification with PEG coatings, ligands, and proteins. In addition, they can be easily broken by heat and bubble generation.

## Experimental

### Materials

L-phosphatidylcholine (PC), cholesterol (Chol), 1,2-distearoyl-sn-glycero-3-phospho- ethanolamine-*N*-[methoxy (polyethylene glycol)-2000] (DSPE-PEG2000), Bionic TM buffer, phosphate buffer saline (PBS), melanin, perfluorohexane (PFH), 5-flurouracil (5-FU), Triton-X, and phosphotungstic acid were obtained from Sigma-Aldrich (St. Louis, MO, USA). Dulbecco’s Modified Eagle’s culture Medium (DMEM), fetal bovine serum (FBS), Roswell Park Memorial Institute Medium (RPMI 1640), and antibiotic antimycotic solution were purchased from WELGENE (Daegu, Korea). All other reagents were used without purification.

### Preparation of perfluorohexane (PFH) emulsion

PFH emulsion was prepared with a previously reported method [24] with some modifications. Briefly, 10 mg of PC was dissolved in 2 ml of ethanol, transferred to a round flask, and vacuum dried. Thereafter, the obtained thin film was hydrated with 110 mM ammonium sulfate buffer. PFH liquid was then added to the flask at a 1:1 ratio of the above solution. To prevent evaporation of PFH, it was stored in an ice-water bath while waiting for the next manufacturing step. To homogenize the emulsion, sonication was performed three times for 30 seconds and cooled in each cycle. Finally, the emulsion solution was extruded 11 times through a 200 nm polycarbonate filter using an Avanti Mini Extruder (Alabaster, AL, USA).

### Preparation of melanin@PFH@5-FU-liposomes

Melanin@PFH@5-FU-liposomes were prepared by the thin film method according to a previously reported method [25]. First, PC (10 mg), Chol (1.5 mg), and DSPE-PEG (3 mg) were dissolved in ethanol. Melanin (1 mg) was dissolved in 1 ml of bioionic buffer. These solutions were mixed in a round flask and sufficiently evaporated under vacuum. The formed thin lipid film was hydrated with a solution of 5-FU (2 mg) dissolved in 2 ml of PBS (pH 7.4). Melanin@5-FU-liposome solution was then sonicated for 9 min (on 3 s, off 1 s) in ice-water conditions using a sonicator (SCIENTZ-IID, SCIENIZ, Zhejiang, China). After sonication, the melanin@5-FU-liposome solution was extruded 11 times through a 200 nm polycarbonate filter using an Avanti Mini Extruder (Alabaster, AL, USA). Free 5-FU was then separated from the melanin@5-FU-liposomes solution using a PD-10 column (PD-10 desalting column, GE Healthcare Life Sciences). Finally, the prepared liposome suspension was mixed with PFH emulsion at a 1:1 ratio and sonicated for 90 seconds. The obtained melanin@PFH@5-FU-liposome was extruded 11 times through a 200 nm filter.

### Characterization

Dynamic light scattering (DLS, Zetasizer NanoZS90, Malvern Instruments Ltd., Worcestershire, United Kingdom) was used to confirm size distributions of various amounts of PFH emulsion and melanin@PFH@5-FU-liposome. The morphology of melanin@PFH@5-FU-liposome was observed using a transmission electron microscope (TEM, H-7500, Hitachi Ltd., Tokyo, Japan). Phosphotungstic acid was used as a negative stain agent for TEM observation.

### Photothermal conversion and bubble generation ability

To evaluate the photothermal conversion ability of melanin@PFH@5-FU-liposome *in vitro*, the solution was transferred to a disposable cuvette and then irradiated with an 808 nm NIR laser at a power density of 1.5 W/cm^2^ for 10 min. An infrared (IR) thermal imaging camera (C2, FLIR System Inc., Sweden) was used to record temperature changes every minute. Distilled water was used as a control group to compare temperature changes. To evaluate bubble generation ability, when melanin@PFH@5-FU-liposome solution was irradiated with an 808 nm NIR laser at an intensity of 1.5 W/cm^2^ for 10 min, bubble generation was visually observed. Specifically, to observe bubble formation, the phantom model used a latex tube with a diameter of 5 mm. The melanin@PFH@5-FU-liposome solution (3 ml) was then transferred to a latex tube. In the same manner as described above, while irradiating melanin@PFH@5-FU-liposome with a laser, bubble generation was observed using an ultrasound imaging system (SONON, B-mode, Healcerion, Seoul, Korea).

### *In vitro* drug release behavior

Drug release behavior of melanin@PFH@5-FU-liposome induced by bubble generation of PFH due to the photothermal effect of melanin was evaluated with or without near-infrared laser irradiation. In detail, melanin@5-FU-liposome and melanin@PFH@5-FU-liposome were transferred to disposable cuvettes and irradiated with an 808 nm NIR laser at a power density of 1.5 W/cm^2^ for 10 min. Thereafter, each solution was placed in a dialysis bag (MWCO 12,000 Da), immersed in 5 ml of PBS and then placed in a water bath adjusted to 37°C. Released medium was collected at predetermined time points. The absorbance of the drug was measured using a UV-spectrophotometer (T60U, PG Instruments Limited, UK) at a wavelength of 265 nm. The concentration of drug released was calculated using a standard calibration curve. To measure drug loading efficiencies of melanin@5-FU-liposome and melanin@PFH@5-FU-liposome, these liposome membranes were disrupted using Triton-X to induce the release of the encapsulated drug. The absorbance of 5-FU was then measured using the UV-spectrophotometer.

### Cytotoxicity and PTT-mediated cell death

HaCaT and CT26 cell lines were used to evaluate the cytotoxicity and chemo-photothermal-mediated cell death effect of melanin@PFH@5-FU-liposome. HaCaT and CT26 cells were cultured in DMEM and RPMI1640 medium containing 10% fetal bovine serum in a CO_2_ cell incubator (37°C, 5% CO_2_), respectively. To evaluate the cytotoxicity of melanin@PFH@5-FU-liposome, cell viability was measured using an MTT method. Briefly, in a 48-well plate, 4×10^4^ cells per well were treated with various amounts of melanin@PFH@5-FU-liposome and cultured in a CO_2_ cell incubator for 24 h. Thereafter, cells were treated with a MTT reagent (40 μl). After incubating 37°C for 3 h, the medium containing the MTT reagent was removed. Finally, the purple crystal product formed inside living cells was dissolved in DMSO and the absorbance of the mixture was measured at 570 nm to calculate cell viability. To confirm chemo-photothermal-mediated cell death, 2×10^5^ cells per well were treated with various amounts of melanin@PFH@5-FU-liposome and then cultured in a CO_2_ cell incubator for 6 h. In the same manner as described above, the medium containing melanin@PFH@5-FU-liposome was removed and replaced with a new medium. Thereafter, the plate was irradiated with an 808 nm laser at an intensity of 1.5 W/cm^2^ for 10 min. After laser irradiation, cells were cultured for 24 h. MTT assay was then performed.

### Tumor therapy *in vivo*

To investigate the tumor therapeutic effect of melanin@PFH@5-FU-liposome, we established a CT26 xenograft tumor model. Mice (male, 6 weeks old, Orient Bio Inc., Seoul, Korea) were anesthetized with isoflurane. Then 0.1 ml of 5×10^5^ CT26 cells suspended in Matrigel was inoculated subcutaneously into each mouse to prepare the animal model. After preparing the mouse tumor model, mice were randomly divided into three groups: (1) control group, (2) melanin@PFH@5-FU-liposome injection group, and (3) melanin@PFH@5-FU-liposome injection with laser irradiation group. First, 200 μl of melanin@PFH@5-FU-liposome was intravenously injected into the tail vein of each mouse in the group corresponding to sample injection. Laser irradiation (808 nm, 1.5 W/cm^2^, 10 min) was performed at 1 h, 4 h, and 12 h after intravenous injection. After laser irradiation, temperature change in the tumor was checked using an IR thermal imaging camera. The presence or absence of bubble formation was observed using a portable ultrasound imaging system. The length and width of the tumor were measured using digital calipers. Tumor volume was calculated according to the following formula: (tumor length × tumor width^2^)/2.

### Histological analysis

Mice were sacrificed at 14 days after treatment procedures in various groups. Major organs (heart, kidney, liver, lung, and spleen) including tumor tissues were removed from mice and stained with hematoxylin and eosin (H&E). Observation of stained tissues was performed using an optical microscope (FX-II, Olympus Inc., Tokyo, Japan).

### Statistical analysis

Statistical comparisons among multiple treatment groups were performed using analysis of variance (ANOVA). Probability (*p*) value less than < 0.05 was regarded as statistically significant. Experimental results are presented as means ± standard deviation (S.D.).

## Results

### Characterization of melanin@PFH@5-FU-liposomes

**Fig. 2:**
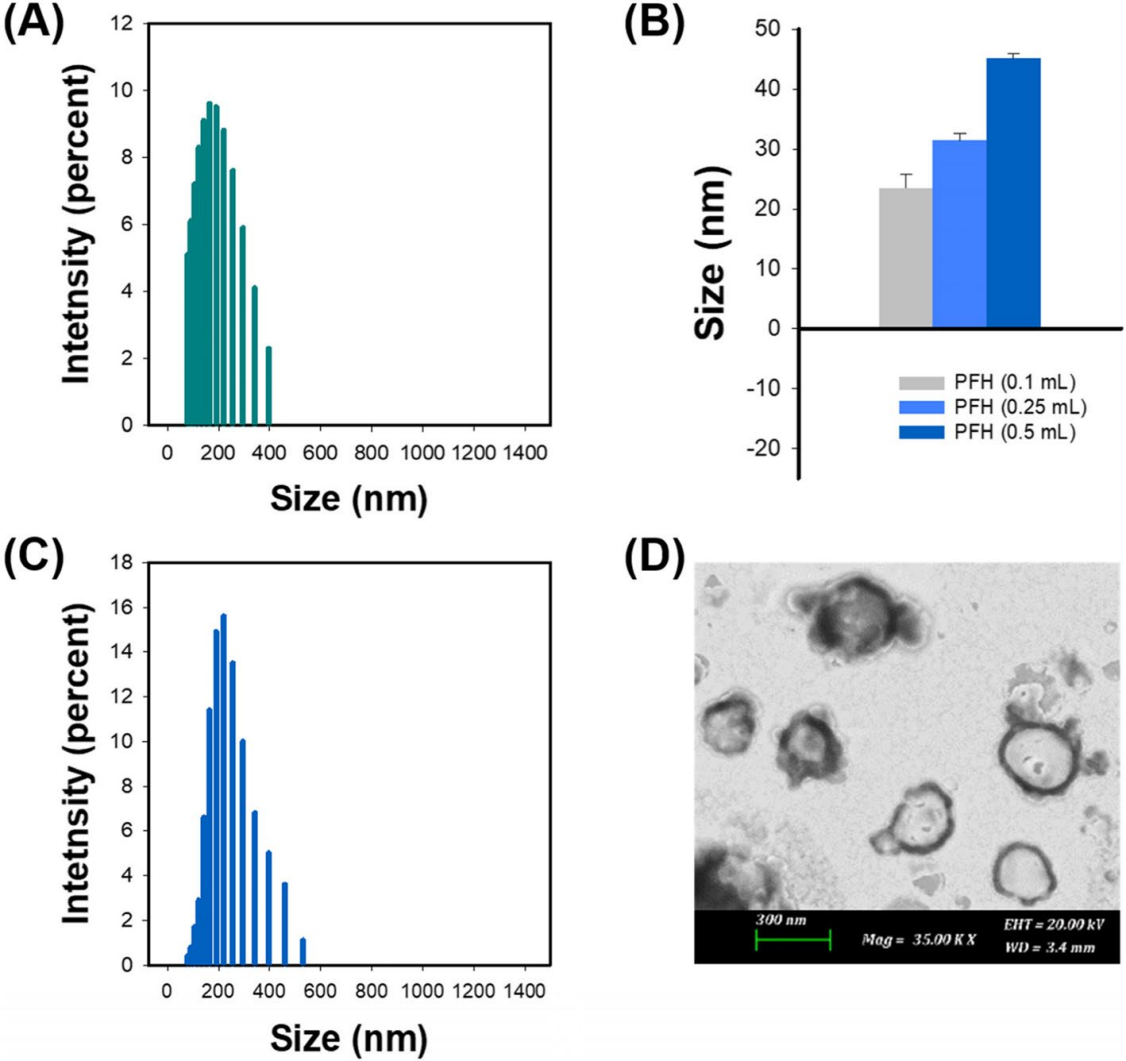
(**A**) Size distribution of melanin@5-FU-liposomes, (**B**) Mean diameter of PFH emulsions, (**C**) Size distribution and (**D**) TEM image of melanin@PFH@5-FU-liposomes

As shown in Fig. 2A, the average size of melanin@5-FU-liposomes without loading of PFH was 148.1 ± 7.5 nm. When the amount of PFH was 0.1, 0.25, or 0.5 ml, corresponding mean size of PFH emulsion was 23.5 ± 2.2 nm, 31.4 ± 1.2 nm, or 45.13 ± 0.86 nm, respectively (Fig. 2B). In further studies, 0.25 ml of PFH was applied and used for preparing melanin@PFH@5-FU-liposomes. The mean diameter of prepared melanin@PFH@5-FU-liposomes was 209.6 ± 4.3 nm (Fig. 2C). In TEM observation (Fig. 2D), it was confirmed that melanin@PFH@5-FU-liposomes had a spherical shape with PFH emulsion inside the liposome. The loading efficiencies of 5-FU and melanin contained within the liposome were 56.3±4.3% and 57.9±0.1%, respectively.

### Light-heat conversion efficiency *in vitro*

**Fig. 3:**
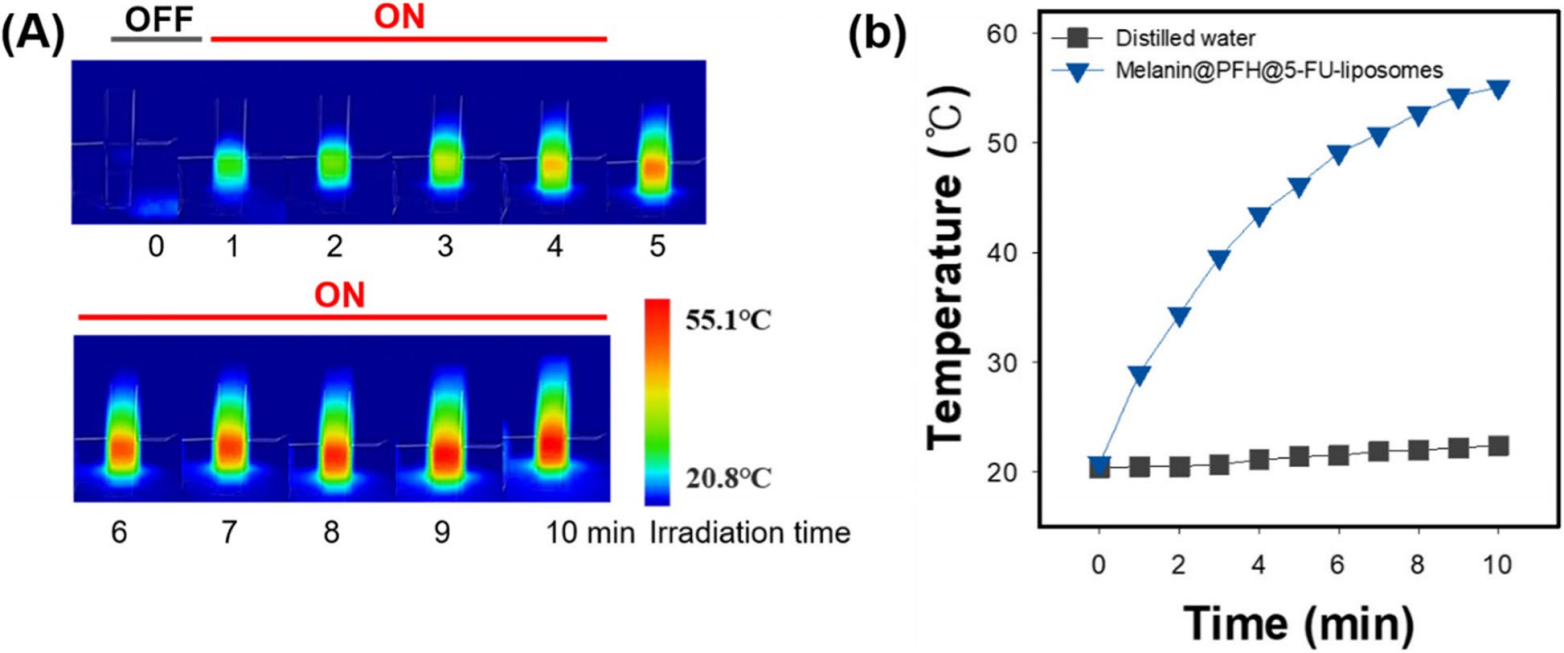
Photothermal conversion ability of melanin@PFH@5-FU-liposomes. (**A**) IR thermal imaging camera images and (**B**) temperature change curve of melanin@PFH@5-FU-liposomes for 10 min at 1.5 W/cm^2^ under NIR 808 nm laser irradiation

To evaluate the light to heat conversion ability of melanin@PFH@5-FU-liposome, temperature increase according to irradiation time with a 808 nm laser was evaluated with a thermal imaging camera (Fig. 3). The control (distilled water) showed a temperature increase of 1.7°C after 10 min of irradiation with the 808 nm laser. On the other hand, the temperature of the melanin@PFH@5-FU-liposome group was increased to 55°C at 10 min after irradiation with the 808 nm laser, showing an increase of 30°C or more compared to the control group.

### Ultrasound imaging of bubble generation

**Fig. 4:**
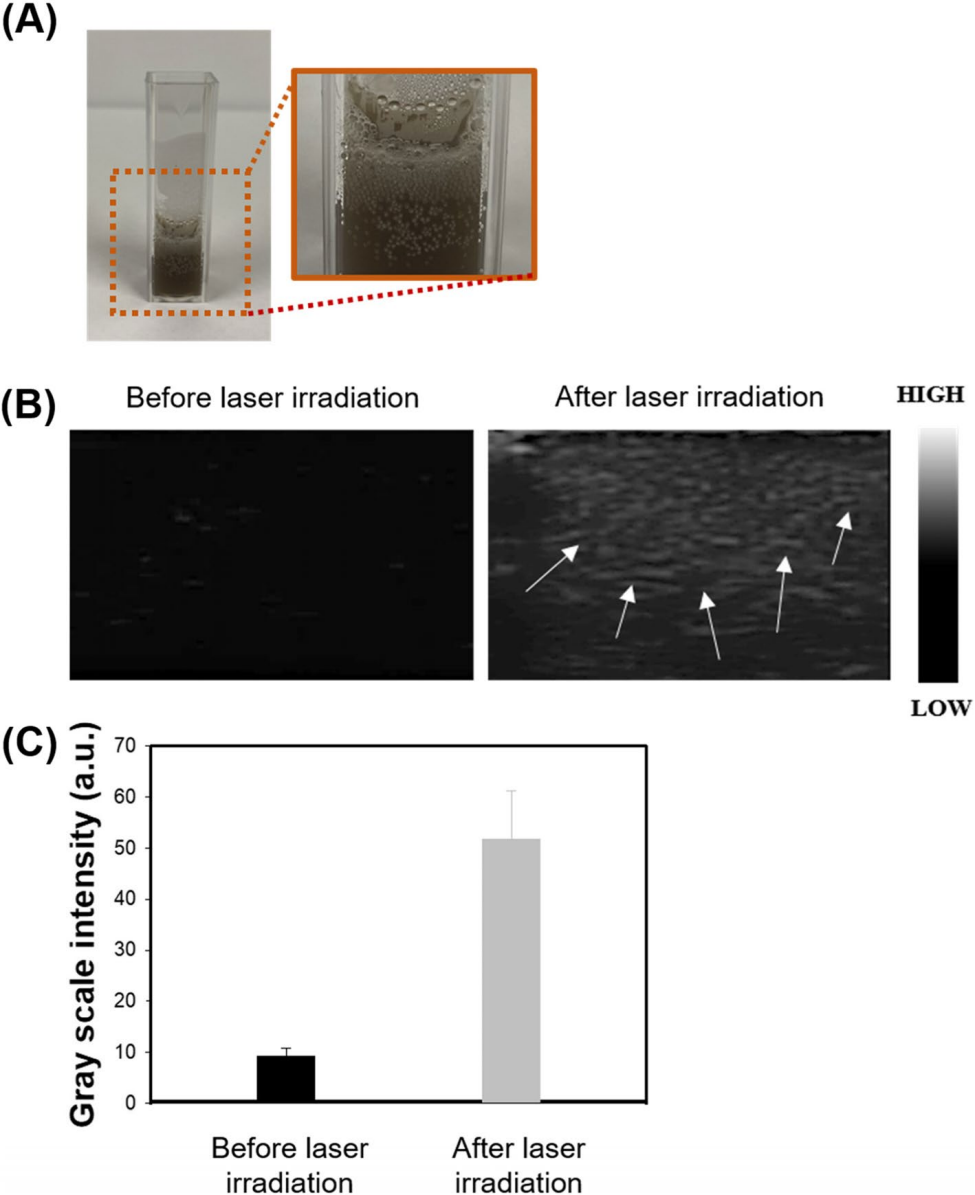
Evaluation of bubble generation ability. (**A**) Photograph, (**B**) ultrasound image, and (**C**) gray scale intensity of bubbles generated in melanin@PFH@5-FU-liposomes by 808 nm NIR laser. White arrows within the ultrasound image indicate bubble generation

To investigate bubble generation by heat of photothermal effect, melanin@PFH@5-FU-liposome solution was transferred to a disposable cuvette and then irradiated with a laser to visually observe the formation of bubbles inside and on the surface of the solution. As a result, as the laser irradiation time increased, the temperature also increased. Bubbles were gradually observed in the solution, but remarkably observed on the surface (Fig. 4A). For a more accurate determination of bubble formation, ultrasound imaging was performed. A latex tube including melanin@PFH@5-FU-liposome solution was used as a phantom model for ultrasound imaging. As shown in Fig. 4B and C, ultrasound imaging contrast enhancement by bubble formation was not observed in the image without laser irradiation. On the other hand, after laser irradiation, PFH phase transition occurred due to temperature rise. Signal of contrast was significantly increased, indicating that bubbles were generated. Generated bubble scattered the ultrasonic wave. During imaging, the bubbles appeared as white as the arrow in the image.

### 5-FU release properties

**Fig. 5:**
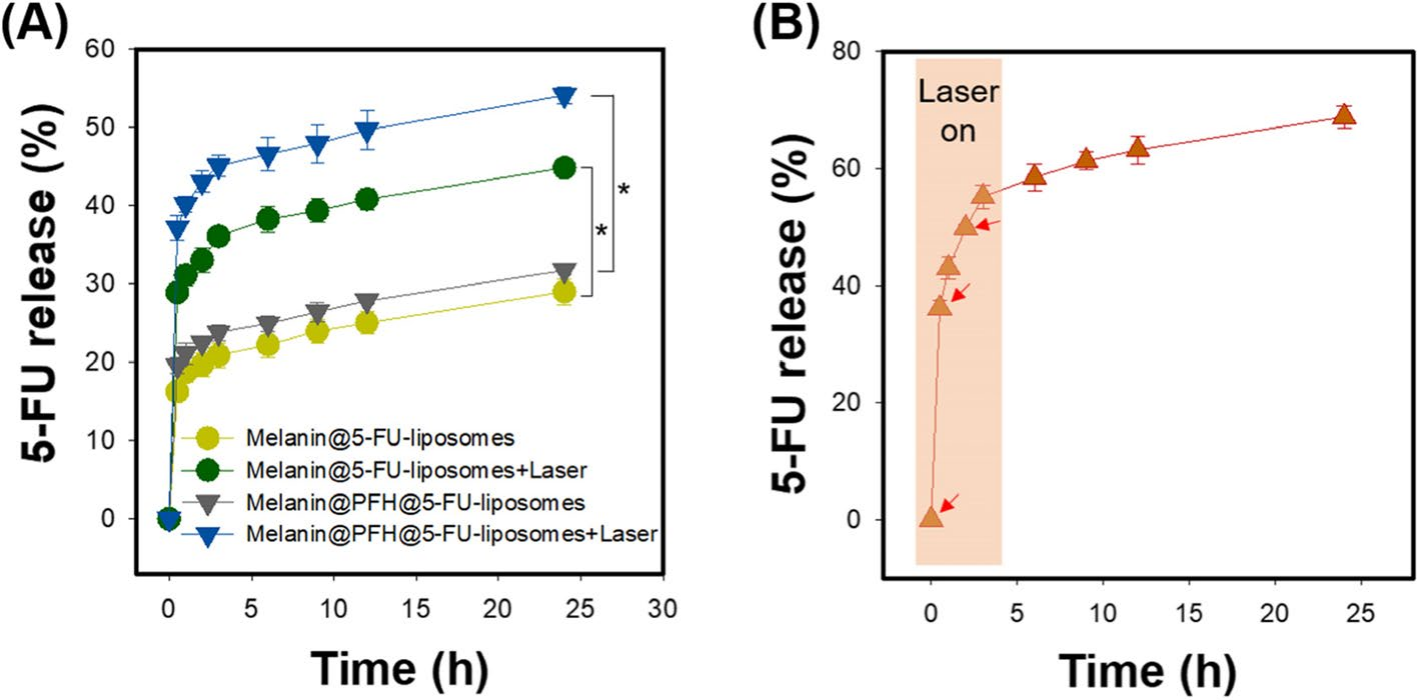
NIR-triggered 5-FU release behavior from melanin@PFH@5-FU-liposomes. (**A**) Release profile of 5-FU from melanin@PFH@5-FU-liposomes with and without PFH and laser irradiation, (**B**) Release profile of 5-FU by repeated laser irradiation

Next, we checked whether release of the drug from the melanin@PFH@5-FU-liposome was induced by the photothermal effect and bubble generation of PFH. Drug release behavior of 5-FU was evaluated using melanin@5-FU-liposome and melanin@PFH@5-FU-liposome with or without PFH and laser irradiation (Fig. 5A). As a result, the release rate of 5-FU was about 16% higher in the melanin@5-FU-liposome group with laser irradiation than that of the non-laser irradiated melanin@5-FU-liposome group. More specifically, melanin@5-FU-liposome without laser irradiation released 29.0 ± 1.6% of the total 5-FU at 24 h of release test time, whereas melanin@5-FU-liposome with laser irradiation released 44.9 ± 1.0% at the same time. Whether drug release was further promoted by bubble formation of PFH sequentially caused by the photothermal effect was then determined. From the melanin@PFH@5-FU-liposome, 31.8 ± 0.5% of the loaded 5-FU was released at 24 h without laser irradiation. Interestingly, melanin@PFH@5-FU-liposome with laser irradiation released 54.1 ± 1.1% of the loaded 5-FU at 24 h. The difference in drug release rate was about 22% between melanin@PFH@5-FU-liposome with and without laser irradiation. To determine whether such a continuous mechanism was caused by laser irradiation, instantaneous drug release was investigated by performing additional irradiation with a laser at 30 min and 2 h after the initial laser irradiation. As shown in Fig. 5B, the release of loaded 5-FU was rapidly and significantly increased at laser irradiation time. The release amount of 5-FU was measured to be 55.2 ± 2.0% of the loaded drug within 3 h, confirming that the drug release was induced by laser irradiation.

### Cell cytotoxicity of melanin@PFH@5-FU-liposome

**Fig. 6:**
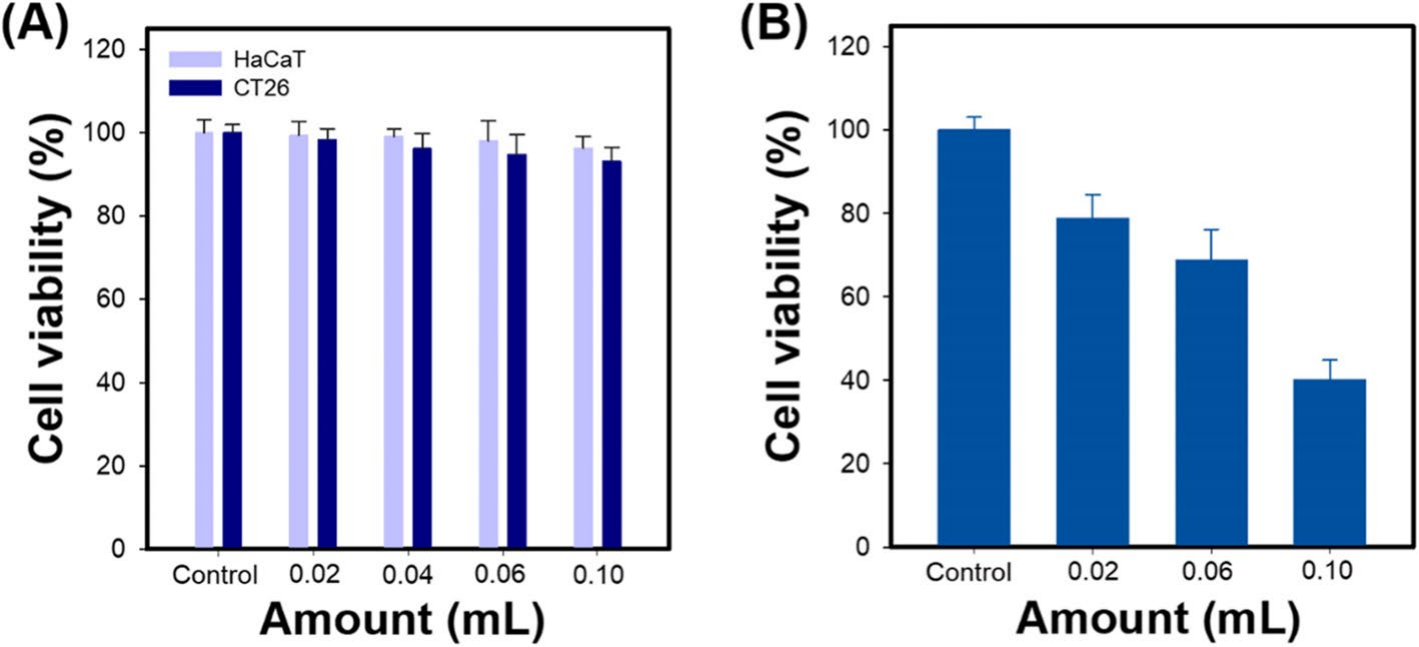
*In vitro* cytotoxicity study. (**A**) Cell viability of HaCaT and CT26 cells treated with melanin@PFH@5-FU-liposomes at various amount and (**B**) Cell viability of CT26 cells treated with melanin@PFH@5-FU-liposomes for 10 min at 1.5 W/cm^2^ under NIR 808 nm laser irradiation

Cytotoxicity of melanin@PFH@5-FU-liposome was evaluated using CT26 cancer cells and HaCa T normal cells. As the amount of melanin@PFH@5-FU-liposome increased without laser irradiation, the viability of both cell lines was maintained above 93% even at high concentrations (Fig. 6A). On the other hand, upon laser irradiation, melanin@PFH@5-FU-liposome was toxic to CT26 cells in a dose-dependent manner. Cell viability was 40.1 ± 4.8% after treatment with melanin@PFH@5-FU-liposome at the highest dose (Fig. 6B).

### Ultrasound imaging and photothermal imaging *in vivo*

**Fig. 7:**
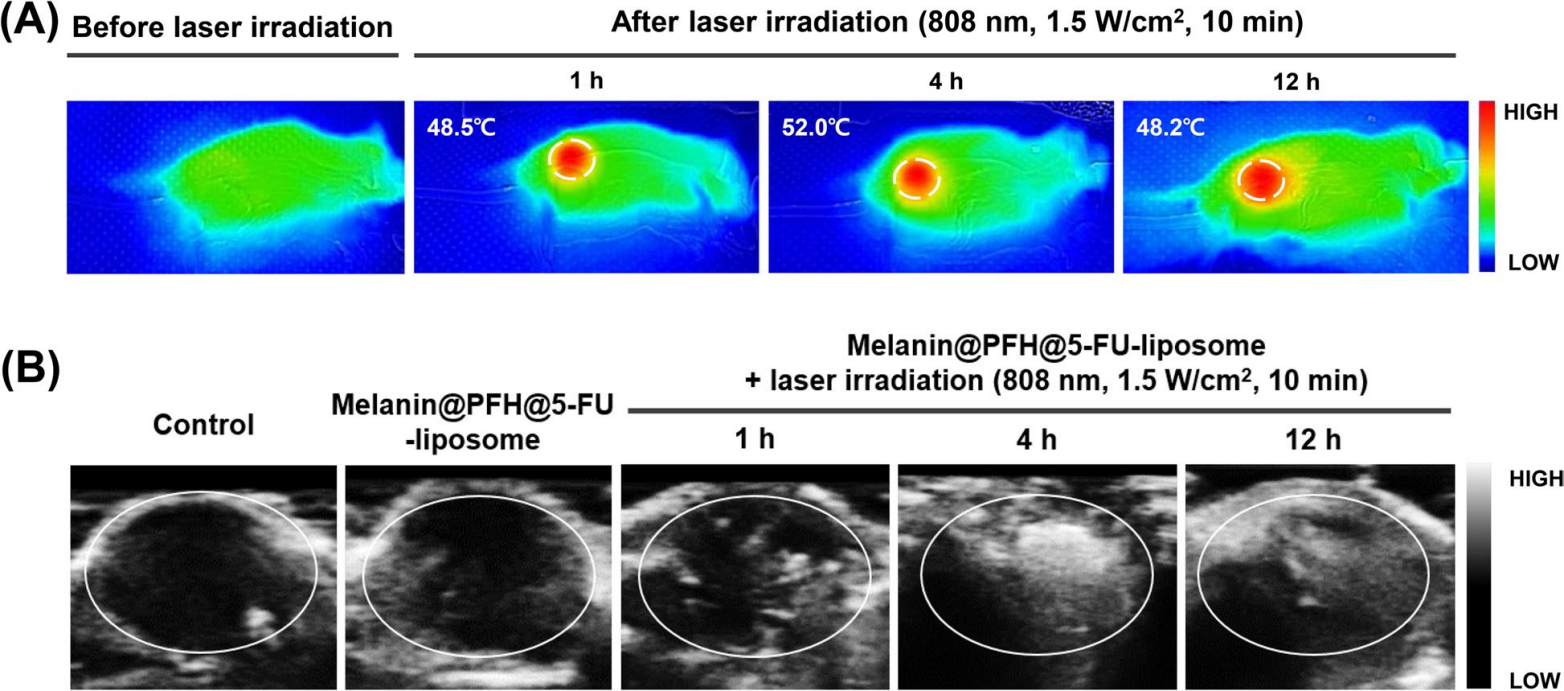
*In vivo* animal study for heat and bubble generation in tumor tissues. (**A**) IR thermal images and (**B**) ultrasound images of tumor tissues in CT26-bearing mice before and after laser irradiation at 1 h, 4 h, and 12 h after intravenous injection of melanin@PFH@5-FU-liposomes

First, we established a colorectal cancer mouse model to confirm the accumulation and temperature increase of melanin@PFH@5-FU-liposome *in vivo* and to acquire ultrasound images due to bubble generation under laser irradiation after intravenous injection of melanin@PFH@5-FU-liposome via mouse tail vein. As shown in Fig. 7A, no temperature increase was observed at the tumor site before laser irradiation. On the other hand, when laser irradiation was performed at 1 h after injection of melanin@PFH@5-FU-liposome, the temperature of the tumor site rose to about 48.5°C. Thereafter, at 4 h and 12 h, temperatures of the tumor site were 52°C and 48.2°C, respectively. At the same time, formation of bubbles in the tumor site due to the increase in temperature was observed. As shown in Fig. 7B, no contrast enhancement in ultrasound imaging due to bubble formation was observed in the control or melanin@PFH@5-FU-liposome without laser irradiation group. On the other hand, when laser irradiation was performed at 1 h, 4 h, and 12 h after injection of melanin@PFH@5-FU-liposomes, contrast signal at the tumor site was significantly enhanced. The highest contrast enhancement by bubbles in tumor site was observed at 4 h after injection.

### Tumor therapeutic effect of melanin@PFH@5-FU-liposome

**Fig. 8:**
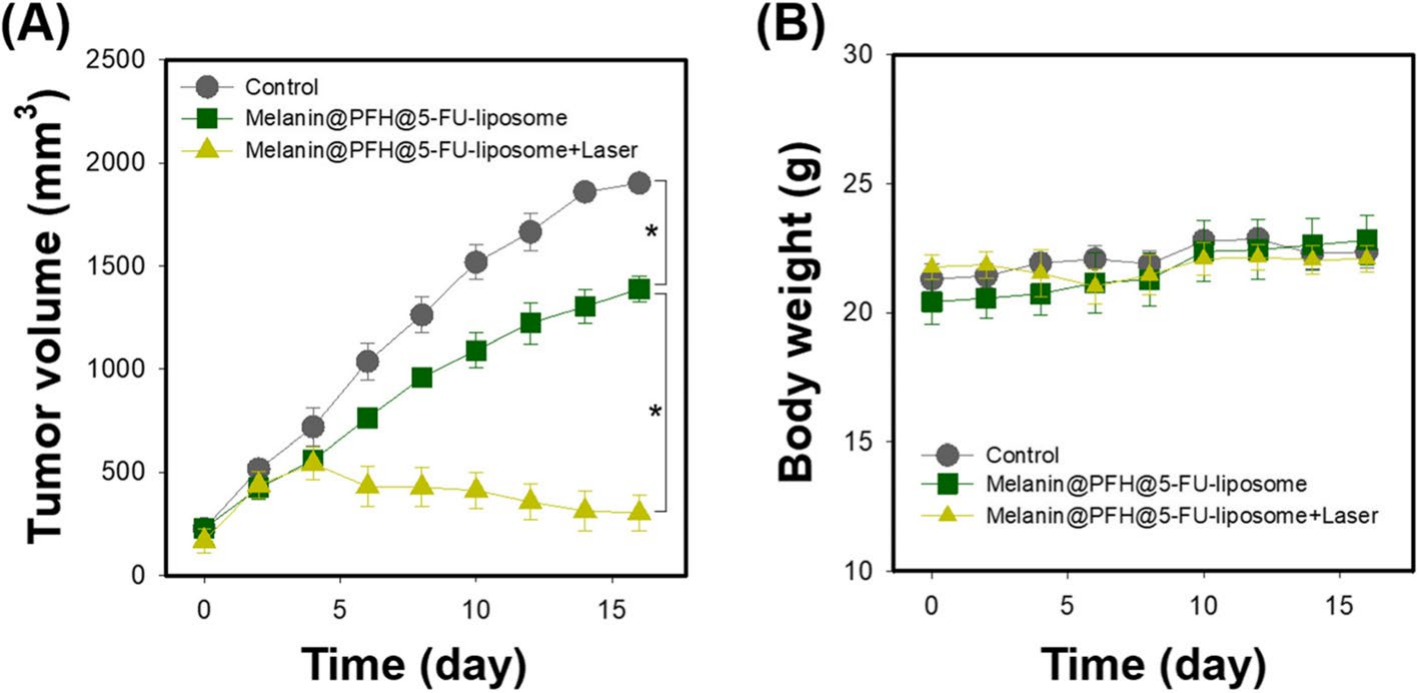
*In vivo* photothermal tumor therapeutic effects of melanin@PFH@5-FU-liposomes. (**A**) Tumor growth and (**B**) body weight changes of CT26-bearing mice after injection of melanin@PFH@5-FU-liposomes without or with laser irradiation for a period of 16 days

**Fig. 9:**
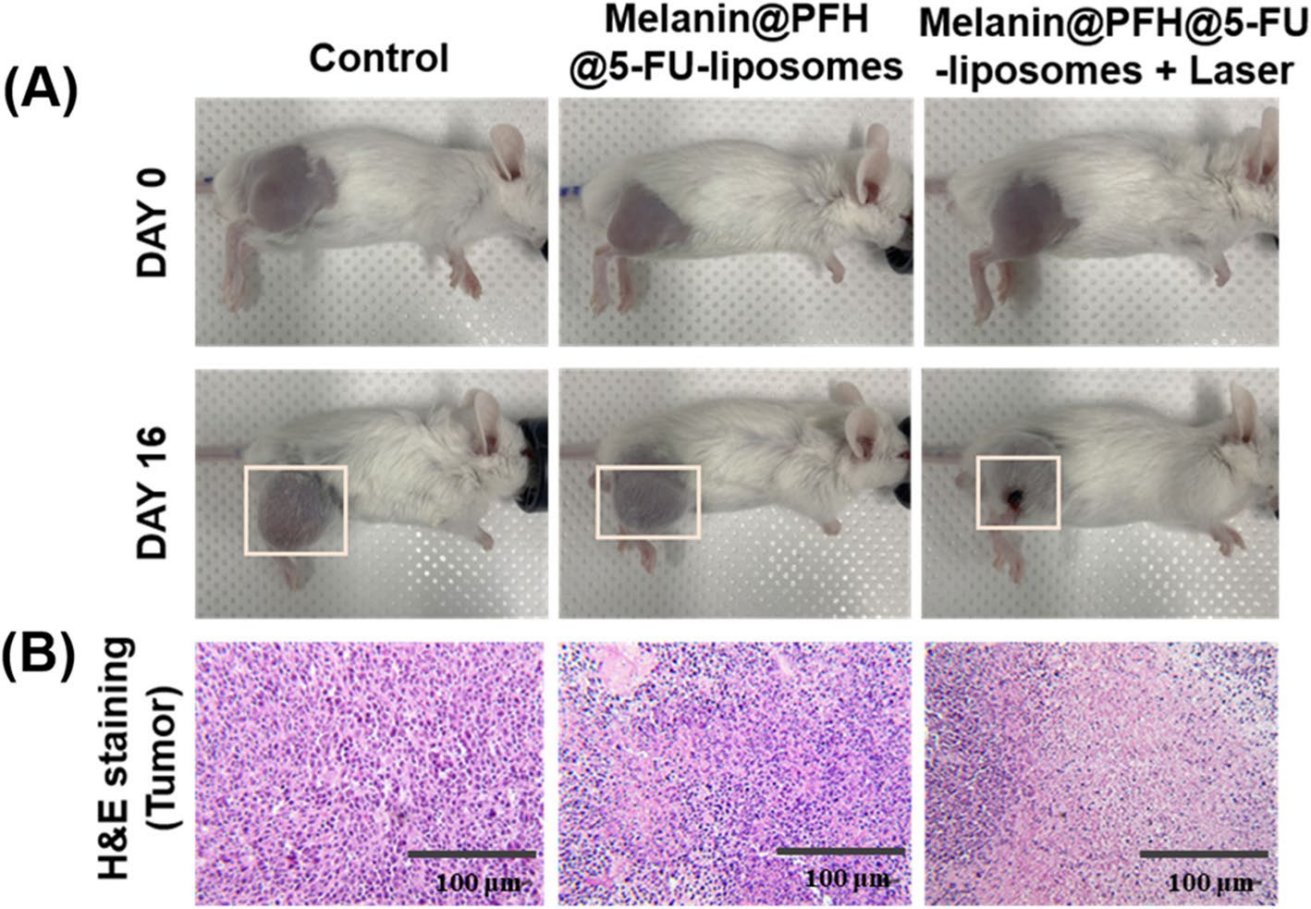
*In vivo* photothermal tumor therapeutic effects. (**A**) Photographs of CT26-bearing mice and (**B**) photo images of H&E stained tumor tissues at 16 days post treatment of laser irradiation following injection of melanin@PFH@5-FU-liposomes

**Fig. 10:**
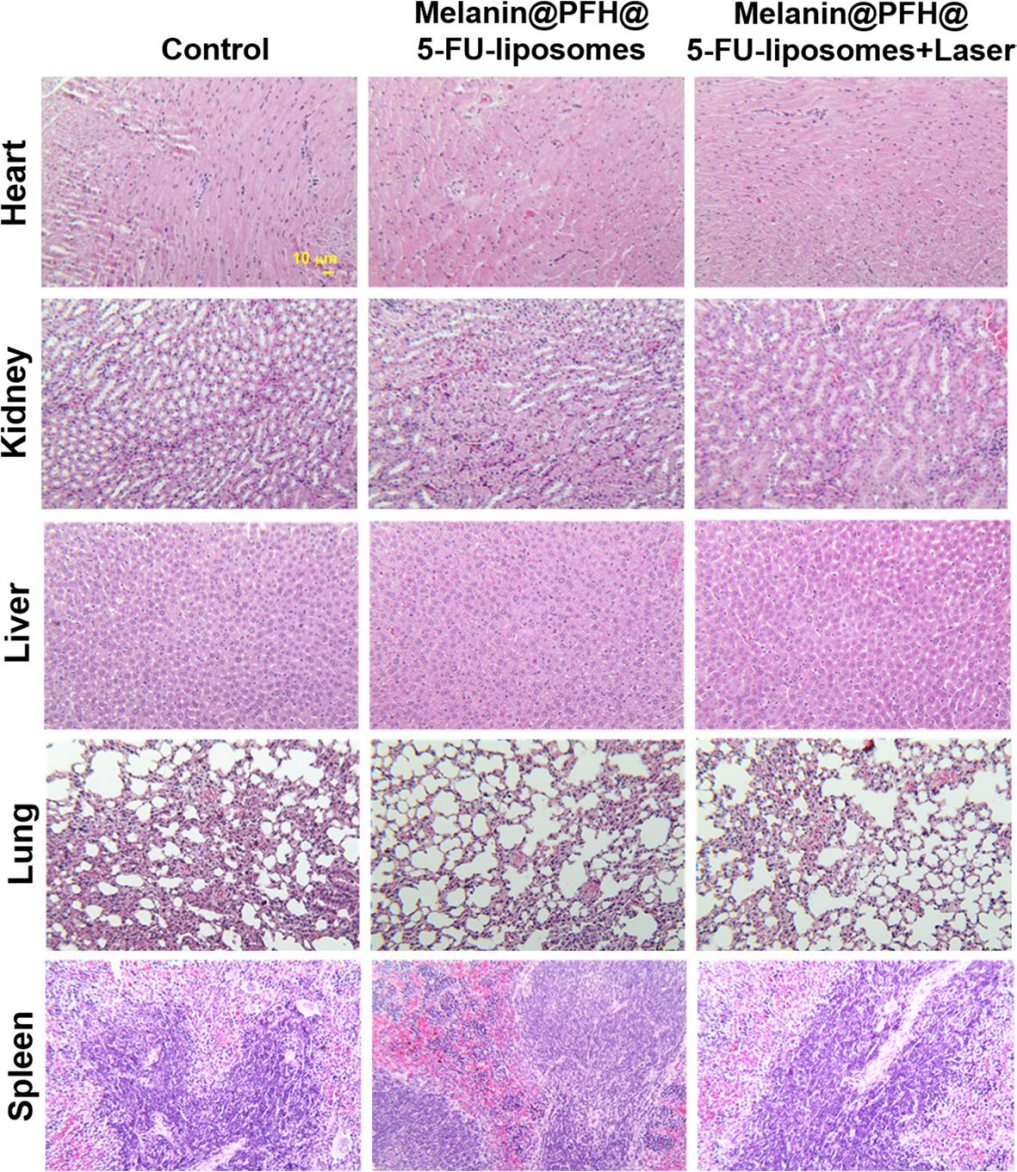
Photo images of H&E stained tissues (heart, kidney, liver, lung, and spleen) collected from mice of different groups

The *in vivo* therapeutic effect of melanin@PFH@5-FU-liposome was evaluated using CT26 tumor-bearing mice. Figure 8a presents mouse tumor sizes in each group measured using calipers for 16 days. Tumor sizes of control mice increased dramatically over time. In mice injected with melanin@PFH@5-FU-liposome without laser irradiation, tumor growth was suppressed than that in control mice (*p < 0.01). On the other hand, tumor sizes slightly increased in the early stage of mice injected with melanin@PFH@5-FU-liposome under laser irradiation. However, tumor sizes gradually decreased over time. Tumor growths in mice injected with melanin@PFH@5-FU-liposome under laser irradiation were inhibited significantly compared to those in other groups (*p < 0.01). In addition, as shown in Fig. 8b, there was no significant change in mouse body weight in any group. Figure 9A shows photographs of all mouse groups at 16 days post treatments. Tumor growths in control mice and mice injected with melanin@PFH@5-FU-liposome were clearly observed at 16 days post treatment. However, in mice injected with melanin@PFH@5-FU-liposome followed by laser irradiation, heat generated by laser irradiation induced necrosis of cancer tissues, resulting in inhibition of tumor growth and the formation of scabs. H&E staining of tumor tissues extracted from mice was performed at 16 days post treatments. As shown in Fig. 9b, no necrotic area was observed in tumor tissues of control mice or mice injected with melanin@PFH@5-FU-liposome. On the other hand, a large area of necrosis was observed in the tumor tissue of mouse injected with melanin@PFH@5-FU-liposome followed by laser irradiation. Histopathological analysis of major organs (heart, kidney, liver, lung, spleen) was performed to observe whether toxicity occurred *in vivo*. In H&E images, there was no damage to normal tissues in any group (Fig. 10).

## Discussion

Melanin has a chromophore that can absorb light and convert it into heat [26]. Due to its excellent biocompatibility and good biodegradability, melanin is attracting attention as an alternative to existing photothermal agents [27]. More importantly, it can effectively generate heat by absorbing NIR light with good tissue penetration, which has potential for *in vivo* applications. Despite these advantages, the use of melanin in biomedical fields is limited because it is soluble only in alkaline solvents, not in physiological solvents [28]. To increase the applicability of melanin in biomedical fields, it is necessary to improve the dispersibility and solubility of melanin in physiological environments [29]. Herein, to broaden biomedical application range of melanin, liposome having hydrophilic and hydrophobic parts was applied to control drug release and therapy tumors. In addition, PFH was incorporated into liposomes for bubble generation utilizing the photothermal effect of melanin. The generated bubble from PFH by heat can disrupt the bilayer of the liposome, releasing the drug inside and enabling ultrasound imaging.

Melanin and PFH were successfully incorporated into liposomes. We have already reported melanin-loaded liposome for photothermal tumor therapy [25, 30]. In the previous our research, the amount of melanin that generated heat necessary for tumor treatment and drug release was confirmed. In the present study, PFH was additionally loaded in the melanin-liposome. Although the ability to generate bubbles was enhanced as the amount of PFH increased, the amount of 0.5 ml was not suitable for being contained in the liposome because the size of the liposome became too large. Therefore, 0.25 ml of PFH was loaded in the liposome. Consequently, based on results of appropriate nanosizing, melanin@PFH@5-FU-liposomes are expected to accumulate in tumor tissues due to enhanced permeability and retention (EPR) effect [31]. After accumulation, when the tumor site was irradiated with the laser, melanin@PFH@5-FU-liposome could generate heat and promote PTT-mediated necrosis of tumor tissues. In addition, the created heat could generate bubbles from PFH in liposomes, resulting in instantaneous drug release and enabling ultrasound imaging. In photo-heat conversion study, the temperature of melanin@PFH@5-FU-liposome increased to 55°C after laser irradiation (Fig. 3). A temperature of 55°C is sufficient to kill cancer cells. It also could cause a phase transition of PFH because PFH undergoes a phase transition from liquid to gas upon reaching 56°C [32]. By using vapor pressure characteristics of various PFC types, it is possible to control the phase transition temperature in a wide range from low to high temperatures. As shown in Fig. 4, when melanin@PFH@5-FU-liposome was laser irradiated, phase transition of PFH occurred due to the photothermal effect. Liquid PFH was converted into bubbles, promoting the release of 5-FU in the liposome by breaking the lipid bilayer. A carrier generating bubbles and containing a drug has a high sensitivity for ultrasound imaging of a lesion and the characteristic of releasing the drug in an instant. Thus, it can be used simultaneously as a therapeutic and diagnostic agent [33–36]. Figure 5 shows that drug release can be induced by the photothermal effect of melanin in response to laser light. In addition, when the liposome containing PFH as laser irradiated, the drug release rate was higher than about 10%. Thus, drug release was induced by bubble formation. Cytotoxicity assessment for biomedical applications is essential. Cancer cells are more sensitive to heat than normal cells, causing apoptosis/necrosis at temperatures above 43°C [37]. As shown in Fig. 6, melanin@PFH@5-FU-liposome had no cytotoxicity without laser irradiation. However, the heat generated from melanin@PFH@5-FU-liposome by exposure to laser irradiation was cytotoxic. *In vitro* cell cytotoxicity results revealed that melanin@PFH@5-FU-liposome was safe for future *in vivo* evaluation. Nano-sized substances can effectively accumulate at tumor sites due to EPR effect [38, 39]. Successful accumulation of melanin@PFH@5-FU-liposome in tumor tissues can be predicted based on local temperature rise of the tumor site when irradiated with a laser after intravenous injection. Although the accumulation of liposomes in the lesion through intravenous injection depends on physicochemical characteristics such as surface charge and particle size, it is the highest after approximately 4 h [40]. In the present study, it was found that melanin@PFH@5-FU-liposome was effectively accumulated in the tumor at 4 h post intravenous injection, showing the highest temperature rise and contrast enhancement of ultrasound images (Fig. 7). Without laser irradiation after injection of melanin@PFH@5-FU-liposome, only the inhibition of tumor growth compared to the control mice by the effect of 5-FU was shown (Fig. 8A). Unlike this, melanin@PFH@5-FU-liposome effectively inhibited tumor growth by inducing drug release and heat generation under NIR laser irradiation. Histological results indicated that melanin@PFH@5-FU-liposome killed tumors under laser irradiation whereas it had no effect on healthy major organs. These results confirmed that melanin@PFH@5-FU-liposome could be used in photothermal tumor therapy and ultrasound imaging.

## Conclusions

In summary, melanin@PFH@5-FU-liposomes were successfully prepared through thin-film hydration. They showed a spherical shape of 209.6 ± 4.3 nm in size. Based on intrinsic photothermal properties of melanin, melanin@PFH@5-FU-liposomes exhibited effective heat generation upon NIR laser irradiation. The light to heat conversion of melanin in the liposome induced vaporization of PFH to create bubbles dramatically. The 5-FU release rate from melanin@PFH@5-FU-liposome was instantaneously increased as a sequential response of photothermal effect. The bubble generation by exposure to NIR laser induced contrast enhancement of ultrasound imaging. The generated heat of melanin@PFH@5-FU-liposome under NIR laser irradiation can induce the death of cancer cells, thereby inhibiting tumor growth effectively. These results demonstrate that melanin@PFH@5-FU-liposome could be used as an attractive agent for both photothermal tumor therapy and ultrasound imaging.

## Data Availability

For data requests, please contact the authors.
